# Physiological Alteration in Sunflower Plants (*Helianthus annuus* L.) Exposed to High CO_2_ and Arbuscular Mycorrhizal Fungi

**DOI:** 10.3390/plants10050937

**Published:** 2021-05-08

**Authors:** Enrique Bellido, Purificación de la Haba, Eloísa Agüera

**Affiliations:** Departament of Botany, Ecology and Plant Physiology, Faculty of Science, University of Córdoba, 14071 Córdoba, Spain; enriquebeag91@gmail.com (E.B.); bv1hahep@uco.es (P.d.l.H.)

**Keywords:** biofertilization, oxidative stress, photosynthetic pigments, nutrients, C/N ratio

## Abstract

Sunflower plants (*Helianthus annuus* L.) in a CO_2_-enriched atmosphere (eCO_2_) were used herein to examine the developmental and physiological effects of biofertilization with mycorrhizae (*Rhizophagus irregularis*). The eCO_2_ environment stimulated colonization using *R. irregularis* mycorrhizal fungi, as compared to plants grown under ambient CO_2_ conditions (aCO_2_). This colonization promotes plant growth due to an increased nutrient content (P, K, Mg, and B), which favors a greater synthesis of photosynthetic pigments. Biofertilized plants (M) under eCO_2_ conditions have a higher concentration of carbon compounds in their leaves, as compared to non-biofertilized eCO_2_ plants (NM). The biofertilization (M) of sunflowers with *R. irregularis* decreased the C/N ratio, as compared to the NM plants, decreasing the hydrogen peroxide content and increasing the antioxidant enzyme activity (catalase and APX). These results suggest that sunflower symbiosis with *R. irregularis* improves the absorption of N, while also decreasing the plant’s oxidative stress. It may be concluded that biofertilization with mycorrhizae (*R. irregularis*) may potentially replace the chemical fertilization of sunflower plants (*H. annuus* L.), resulting in more environmentally friendly agricultural practices. This information is essential to our understanding of the mechanisms influencing the C and N dynamic in future climate change scenarios, in which high CO_2_ levels are expected.

## 1. Introduction

Sunflowers are the fourth most important oilseed crop in the world. They have a high tolerance to drought, making them an ideal alternative for producers in semiarid regions [1]. Yield decrease in sunflowers is due mainly to biotic and abiotic stress factors. The breeding of new cultivars that are resistant to stress factors is a priority for both conventional and modern (biotechnological) breeding.

One of the main problems faced by farmers today is decreased production due to damage caused by natural alterations to the environment. Recently, this problem has been exacerbated by climate change, causing major modifications to ecosystems as a result of extreme climate-related phenomena, such as droughts, snowstorms, floods, heat waves, cyclones, and so forth. [2]. These phenomena lead to stressful situations for plants, resulting in millions of euros of losses every year, as approximately 50% of the annual crop production potential is lost [2,3].

The continuous emission of greenhouse gases (GHGs), mainly CO_2_, is a major cause of climate change, resulting in planetary temperatures that have increased from 2.5 to 7 °C during this century [4]. The anticipated effects of this increase make it necessary to improve our knowledge of the effects of high CO_2_ on plant metabolism during distinct developmental phases. This knowledge will allow for the development of tools and strategies that may improve the adaptive capacity of plants under unfavorable environmental conditions, increasing their production while reducing environmental costs [5].

The use of nitrogenous fertilizers on crops also results in an increase in GHGs such as NxO, thus contributing to environmental contamination [6]. Biofertilizers may be a relevant strategy to combat the environmental impact of nitrogenous fertilizers. Biofertilizers are preparations containing microorganisms (fungi, bacteria, and algae) that are applied to the soil or directly to the plant’s root in order to act as a full or partial substitute for chemical fertilizers [7,8]. They offer beneficial effects for the plant’s growth [9,10].

Biofertilization with fungi is based on the mutualistic and symbiotic associations of fungi with certain plant roots, specifically the so-called mycorrhizae. Over 90% of all plant species make these associations, which offer benefits to the plant as well as the fungi [10,11,12]. Fungi may be classified into three groups: endomycorrhiza, ectomycorrhiza, and ectendomycorrhiza. Endomycorrhiza are responsible for the formation of arbuscular mycorrhizae (AM) [10]. The symbiosis of plants with AM fungi is well known and quite extensive, occurring in 85% of all land plant species [10,13]. This symbiosis is known to have numerous benefits [14], including the mitigation of abiotic stress in plants [15,16].

AM play a fundamental role in the global carbon cycle [17,18], since they can use up to 20% of the plant’s photoassimilates under ambient CO_2_ conditions [19], and they slowly deposit organic compounds such as chitin, glomalin, and other organic materials that protect plants, promoting soil aggregate formation [20]. A high CO_2_ level stimulates the use of photoassimilates by the AM, favoring their growth [21]. This suggests that, in terms of the soil, a higher level of carbon sequestration may be achieved through mycorrhizae symbiosis in future scenarios of high levels of CO_2_ [22]. Most studies to date have indicated that high CO_2_ levels will lead to increased mycorrhizae colonization, as well as changes in arbuscular mycorrhizae communities [23,24].

Therefore, the main objective of this study is to use distinct analytical techniques to determine whether sunflowers (*Helianthus annuus* L.) that have been biofertilized with fungi (*Rizophagus irregularis*) may partially substitute chemical fertilizers to achieve an optimal C/N ratio in plants that have grown in different environmental conditions and which have been altered by climate change. Prior studies have revealed that high levels of atmospheric CO2 and elevated temperatures [25,26,27] may alter the C/N ratio in plants, thereby inducing the senescence process. As such, achieving a balanced C/N ratio through biofertilization with mycorrhizae may reduce the need for chemical fertilization, resulting in more environmentally friendly agricultural practices.

## 2. Results

### 2.1. Mycorrhizal Colonization and Growth Parameters

First, we determined the percentage of successful *R. irregularis* colonization in the sunflower (*H. annuus* L.) roots in distinct treatments. In this study, no mycorrhizal colonization was observed in sunflowers that were not provided with AM inoculum. The inoculated sunflower plants showed 40% and 48% mycorrhizal root colonization under aCO_2_ and eCO_2_ conditions, respectively (Table 1). Plants grown under eCO_2_ conditions had a higher biomass than those grown under aCO_2_ conditions. Biofertilization with *R. irregularis* increased the leaf and stem dry weight and leaf area in both CO_2_ treatments (aCO_2_ and eCO_2_) (Table 1).

### 2.2. Leaf Sugar, Protein, C-N Content, and Nutrient Accumulation 

In the sunflowers, was observed that when grown under eCO_2_ conditions, the content of starch and total soluble sugars (TSS) increased as compared to plants grown under aCO_2_ conditions. Moreover, when fertilized with *R. irregularis,* their carbon compound levels increased in both treatments (eCO_2_ and aCO_2_). On the other hand, we also verified that mycorrhizal symbiosis increased the total protein concentration in the leaves for both the aCO_2_ and eCO_2_ conditions (Table 2). In sunflower leaves, we observed that the percentage of C in the leaf (leaf C%) did not vary between treatments; however, the percentage of N in the leaf (leaf N%) was lower in plants grown under eCO_2_ conditions, although biofertilization with *R. irregularis* increased the percentage of N in the plant. As a result, the C/N ratio was higher in plants grown under eCO_2_ conditions, especially NM plants (Table 2). 

The nutrient content (P, K, Mg, Fe, and B) was determined in plant leaves grown under eCO_2_ and aCO_2_ conditions (N and NM). Levels of P, K, Mg, and B were higher in the plant leaves that were grown under eCO_2_ conditions, as compared to those grown under aCO_2_ conditions. An additive effect was observed in the mycorrhized plants in the uptake of these nutrients. However, no variations in Fe content were observed (Table 3).

### 2.3. Photosynthetic Pigments

The plants grown under eCO_2_ conditions had lower chlorophyll a and b and carotenoid content than those grown under aCO_2_ conditions. However, biofertilization with *R. irregularis* increased the photosynthetic pigment content in both CO_2_ treatments (aCO_2_ and eCO_2_). We observed that, when biofertilized (M), the total chlorophyll content increased by 19.3% in plants grown under aCO_2_ conditions and by 61.6% in plants grown under eCO_2_ conditions (Table 4).

### 2.4. Enzyme Activities of Antioxidant Systems and Hydrogen Peroxide Content

**Figure 1:**
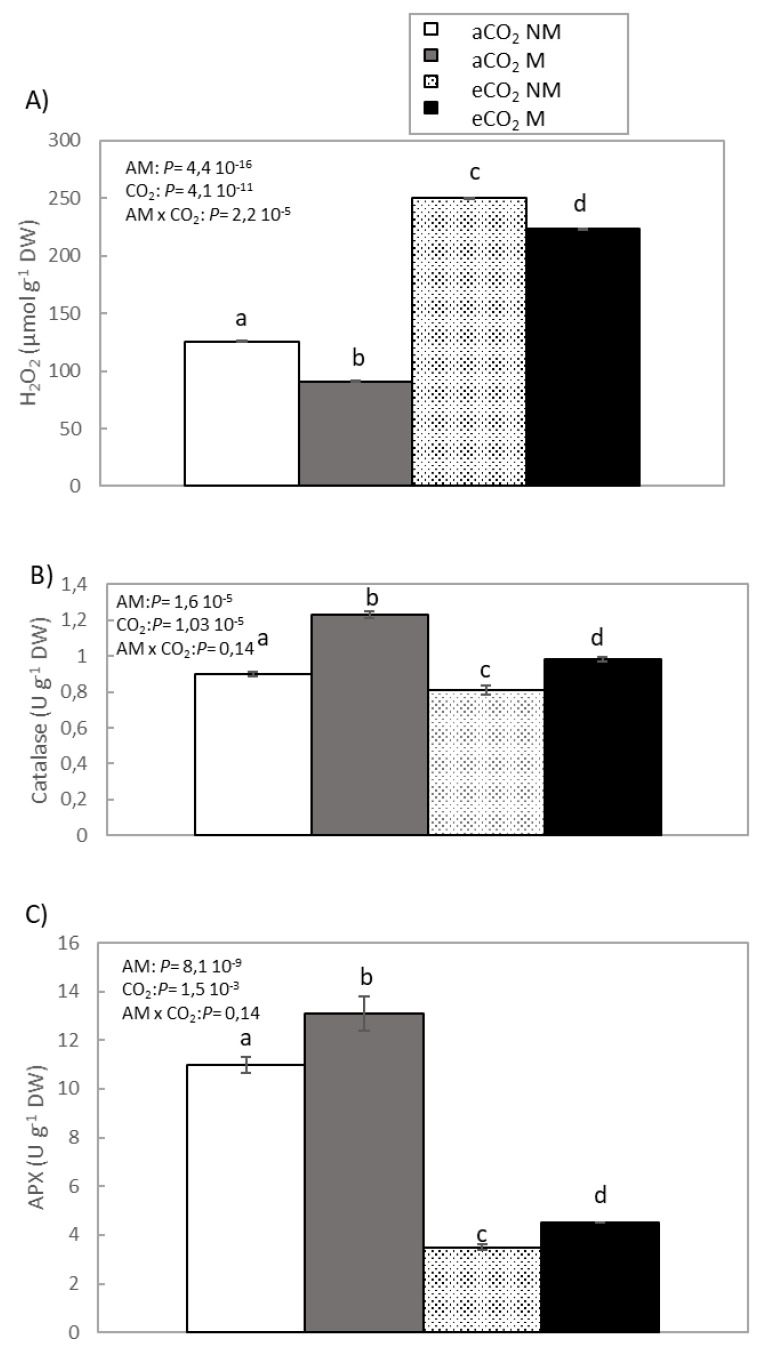
H_2_O_2_ content (**A**), catalase (**B**) and APX (**C**) activities in leaves of sunflower inoculated (M) or not inoculated (NM) with *R. irregularis* and ambient (aCO_2_) and elevated CO_2_ treatments (eCO_2_). Data are means ± SE. Different letters show significant difference among the treatments according to Tukey’s test (*p* 0.05). ** *p* < 0.01, * *p* < 0.05, *NS* = not significant.

H_2_O_2_ content, antioxidant enzyme activity, catalase, and ascorbate peroxidase (APX) in sunflower plant leaves were examined under eCO_2_ and aCO_2_ conditions for both mycorrhized and non-mycorrhized plants. Figure 1 shows that sunflower plants grown under eCO2 conditions had higher levels of H_2_O_2_ than those grown under aCO_2_ conditions. However, when the plants were inoculated with AM, the hydrogen peroxide levels decreased by 27% in the aCO_2_ plants and by 10% in the eCO_2_ plants. We also noted an increase in catalase and peroxidase activities, as compared to non-mycorrhized plants, for both CO_2_ treatments (aCO_2_ and eCO_2_). 

## 3. Discussion

To verify how biofertilization with mycorrhizae (*R. irregularis*) physiologically and metabolically affects sunflower plants (*H. annuus* L.) in a CO_2_-enriched atmosphere, first, the percentage of successful root colonization by the AM fungal (*R. irregularis*) was determined. Table 1 shows that it increased under elevated atmospheric CO_2_ concentrations. This is in line with results found for other plants; most studies have indicated that a high level of CO_2_ leads to increased mycorrhizae colonization, in addition to changes in the arbuscular mycorrhizae communities [20,23,24,28,29]. The increased AM fungi colonization in plants grown under elevated CO_2_ conditions may have been due to an increased plant demand for nutrients and enlarged root biomass caused by greater C assimilation [24]. However, some studies have reported no response or even a decrease in root AM levels in plants grown under high CO_2_ concentrations [12,28]. The extent to which eCO_2_ impacts the crop–AM association remains unclear [10,16]. It has been proposed that differences in AM fungi, plant species, and experiment duration could have caused these discrepancies [11,24]. Table 1 also shows that the leaf dry weight, stem dry weight, and leaf area values for the sunflowers increased under eCO_2_ conditions, with biofertilization having an additive effect on the biomass increase. It has been reported that AM fungi can promote plant growth under eCO_2_ conditions, due to the enhanced nutrient uptake and improved photosynthetic rate of the host plant. Therefore, AM fungi have more photoassimilates from the host plant than under aCO_2_ conditions, resulting in the increased growth of extra-radical hyphae and greater mycorrhizal respiration [30]. 

Sunflower plants grown under eCO_2_ conditions (NM) demonstrated a significant increase in starch and soluble sugar content, as well as in the C/N ratio (Table 2). These results are consistent with previous research on sunflower [26] and soybean [31] plants grown in high CO_2_ environments. Although it was assumed that, initially, some species would require greater assimilation of nitrogen to maintain growth under elevated CO_2_ conditions [32], it has been revealed that under these growth conditions, nitrate assimilation by the plant decreases. This has been verified in sunflower plants, and appears to be the result of the effect on key enzymes in the metabolism of nitrogen, both at a transcriptional (glutamine synthetase 1-GS1and glutamine synthetase 2-GS2) and a post-translational (nitrate reductase-NR, GS and glutamate dehydrogenase-GDH) level [27]. When sunflower plants grown under eCO_2_ and aCO_2_ conditions were inoculated with *R. irregularis* (Table 2), it was verified that colonization increased the starch and soluble sugar levels. However, a lower C/N ratio was observed. Therefore, it is evident that colonization by *R. irregularis* improves the content of nitrogen in the sunflower plant leaf (*H. annuus* L.). An increased nitrogen content has also been observed in plants grown under high CO_2_ levels, and those mycorrhized with AM fungi [30,31]. These results indicate that biofertilization with mycorrhizae (*R. irregularis*) may replace the chemical fertilization of sunflower plants (*H. annuus* L.), leading to more environmentally friendly agricultural practices. This symbiosis may mitigate the effects of climate change, decreasing the excess of CO_2_ and preventing nitrogen from being a limiting factor with regard to plant growth [33].

In sunflower plant leaves grown under eCO_2_ conditions (NM) (Table 3), we found that nutrient uptake (P, K, Mg, and B) significantly increased as compared to plants grown under aCO_2_ conditions (NM). However, no variations were observed in Fe content. Bagheri et al. [34] observed a positive role of AM symbiosis in Zn and Mn uptake, while the acquisition of immobile metal nutrients (Fe and Cu) was not affected. Recently, it has been demonstrated that, in sunflower plants [35] grown in mediums with deficient Fe, mycorrhization with AM mitigates the deficiency symptoms through increased efficiency of H^+^-ATPase activity. When the plants are biofertilized with *R. irregularis*, nutrient availability improves, since AM promotes plant rooting due to the improved development of the root system, thereby increasing water and nutrient uptake [36,37]. The high ambient CO_2_ level improves plant growth, but limitations of P lead to decreased growth, especially of the stems. Both factors, however, when applied individually, increase root growth and exudation, and promote mycorrhizae association [38]. Mycorrhizae not only promote plant growth, since they provide the essential nutrients P, K, Mg, and B, but also protect plants from stressful environmental conditions [39].

De la Mata et al. [26] demonstrated that when sunflower plants were grown under high CO_2_ conditions, the content of photosynthetic pigments (chlorophyll a and b and carotenoids) decreased as compared to plants grown under ambient CO_2_ conditions, as we have verified in this study (Table 4). However, the significant increase in chlorophyll levels that has been found in mycorrhized plants (aCO_2_ and eCO_2_) may be related to the increased P and Mg uptake observed in these plants (Table 3), as reported by Lin et al. [40] in *Leymius chinensis* seedlings. On the other hand, it is also known that the mycorrhization of plants leads to a segregation of substances such as cytokinins, which favor the development of chloroplasts and increase chlorophyll levels [29,41]. Carotenoids act as light-harvesting pigments and play a major role in protecting chlorophyll and membranes from destruction by quenching triplet chlorophyll and removing oxygen from the excited chlorophyll–oxygen complex [42]. Therefore, the reduction in carotenoids may have major consequences in terms of chlorophyll.

ROS are continuously produced unintentionally in plants by means of various metabolic reactions, and plant cells are equipped with antioxidants and scavenging enzymes to keep them low under normal growth conditions [43]. Plants have evolved an elaborate antioxidant system which helps to scavenge endogenously produced ROS [44]. The scavenging of ROS is achieved through the action of different enzymatic and nonenzymatic compounds, including catalase, APX, superoxide dismutase, glutathione reductase, and the enzymes of the ascorbate–glutathione pathway. Nonenzymatic mechanisms include compounds, such as glutathione, ascorbic acid and α-tocopherol, capable of directly scavenging several ROS [45]. In sunflower plants under eCO_2_ conditions (M and NM), oxidative stress was increased, favoring the production of ROS, as observed by the high level of hydrogen peroxide contained in the leaves compared to plants grown under aCO_2_ conditions (M and NM). However, when the plants were inoculated with *R. irregularis*, a significant decrease in hydrogen peroxide content was observed, and there was an increase in the levels of catalase and APX (eCO_2_ and aCO_2_) (Figure 1). The decrease in catalase and APX activities in eCO_2_ plants may it be related with the observed reduction in the N level (Table 2) [26]. In *Arabidopsis* and soybean [46] plants, it has been shown that elevated CO_2_ causes oxidative stress as a result of the increase in protein carbonylation. Plants have developed various antioxidative strategies to flush out these toxic components. The enhancement of antioxidant defenses increases tolerance to different abiotic factors [47]. The AM plants showed reduced hydrogen peroxide levels, thus demonstrating their ability to counteract damage [48]. Chen et al. [49] verified that AM symbiosis in *Zea mays* L. could decrease the accumulation of ROS and reduce the damage of oxidative stress. The activities of catalase and peroxidase of AM inoculated maize were higher than those of non-AM plants. In *Digitaria eriantha* plants that were subjected to abiotic stress, arbuscular mycorrhizal symbiosis regulated physiology and performance under these conditions, with antioxidants and jasmonates participating in this process. AM plants consistently showed higher catalase and APX activity [47]. AM has demonstrated that not only do they confer greater resistance in plants in the face of abiotic and biotic stress, but they also supply plants with the capacity to recover from the negative impacts of adverse phenomena. Mycorrhizae have been identified as significant anti-stress agents in agricultural systems [50].

## 4. Materials and Methods

This work examined modifications in the development and metabolism of sunflowers (*H. annuus* L.) grown under enriched CO_2_ conditions (eCO_2_) and inoculated with AM fungi (*R. irregularis*) (M), as compared to control sunflowers grown under eCO_2_ conditions with no inoculation (NM). We also used control plants grown under ambient CO_2_ conditions (aCO_2_) with (M) and without (NM) inoculation. Sunflower (*H. annuus* L.) seeds from the isogenic cultivar HA-89 (Semillas Cargill, SA, Seville, Spain) were surface-sterilized in 1% (*v/v*) hypochlorite solution for 15 min. After rinsing in distilled water, the seeds were imbibed for 3 h and subsequently sown in plastic trays containing a 1:1 (*v/v*) mixture of perlite and vermiculite, until root emergence. Then, they were moved to pots containing a sterile substrate made of peat (pH 6)/washed sand/vermiculite (1/1.5/1.5) (*v/v/v*) [29]. The pot experiment was carried out under both aCO_2_ (400 μL/L) and eCO2 (800 μL/L) conditions. Plants under aCO_2_ and eCO_2_ conditions were inoculated with 1 g of *R. irregularis* sp. inoculum (Symbiom, 60,000 spores per 60 g) (M) or with 1 g of compost without spores (AMF-Free Carrier Symbiom) (NM). The seeds were germinated and plants were grown in controlled-environment cabinets (Sanyo Gallenkam Fitotron, Leicester, UK) fitted with an ADC 2000 CO_2_ gas monitor with a 16-h photoperiod (400 µmol/m^2^/s) of photosynthetically active radiation supplied by “cool white” fluorescent lamps, supplemented by incandescent bulbs, and a day/night regime of 25/19 °C and 70/80% relative humidity. The plants were irrigated daily with water. Samples of leaves (aged 48 days) were collected 2 h after the onset of the photoperiod. Whole leaves were excised and pooled in two groups. One group was used to take growth parameters and nutrient analyses. The other group was immediately frozen in liquid nitrogen and stored at –80 °C. The frozen plant material was ground in a mortar pre-cooled with liquid N_2_, and the resulting powder was distributed into small vials that were stored at –80 °C until the enzyme activity and metabolite were quantified.

### 4.1. Mycorrhizal Analyses 

For the measurement of mycorrhizal colonization, a fraction of the roots was carefully washed, cut into 1 cm segments, cleared in 10% KOH at 90 °C for 20 min, acidified in 2% HCI for 5 min, and stained with 0.01% acid fuchsin in lactophenol [51]. Mycorrhizal colonization was determined via microscopic calculation. The results are expressed as the percentage of successfully colonized root segments [52].

### 4.2. Growth Parameters

Leaf and stem dry weight was determined after drying the plant material in the oven at 80 °C until the weight was constant. Leaf area (image analysis software, Image-Pro Plus) measurements were taken.

### 4.3. Nutrient Analysis in Leaves

A total of 100 g of leaves were collected and dried at 60 °C for 24 h, and subsequently finely powdered. An analysis of total C and N was performed in a EuroVector EA 3000 elemental analyzer (EuroVector SpA, Via Tortona 5, Milan, Italy) with an automatic injector, provided by the NIR/MIR Spectroscopy Unit at the University of Cordoba, which is part of the Central Service for Research Support (SCAI). The analyses of the remaining elements (P, K, Mg, Fe, and B) were performed by digestion with HNO3/H_2_O2 in an UltraCLAVE Microwave. P concentration was determined by the molybdate blue method, using spectrophotometry [53]. B and Fe were determined by atomic absorption spectrometry. K and Mg concentrations were determined by flame photometry. 

### 4.4. Biochemical Determinations in Leaves

Frozen material was homogenized with cold extraction medium (4 mL/g) consisting of 50 mM Hepes-KOH (pH 7), 5 mM MgCl2, and 1 mM EDTA. The homogenates were filtered through four layers of cheesecloth, and centrifuged at 28,710 g at 4 °C for 15 min. The supernatant was collected and stored at 4 °C for total soluble sugar (TSS) and total soluble protein (TSP) determinations. The pellet was used to determine starch, which was estimated after an iodine reaction [54]. Leaf TSS was analyzed with the anthrone reagent method. Absorbance was measured using a spectrophotometer at 620 nm [55]. Leaf TPS was measured with the Bradford protein dye-binding method [56], using bovine serum albumin (BSA). Pigments were determined in plant extracts by HPLC, according to the method of Cabello et al. [57]. 

### 4.5. H_2_O_2_ Determination, Extraction, and Assay of Antioxidant Enzymes in Leaves

For H_2_O_2_ determination, 1 g of leaf material was ground with 10 mL of cold acetone in a cold room and passed through Whatman filter paper. H_2_O_2_ was determined by the formation of the titanium–hydroperoxide complex, according to the method of Mukherjee and Choudhuri [58].

Enzyme extracts were prepared by freezing a weighed amount of leaf samples in liquid nitrogen to prevent proteolytic activity, followed by grinding in a 0.1 M phosphate buffer at pH 7.5 containing 0.5 mM EDTA and 1 mM ascorbic acid at a 1:10 (*w*/*v*) ratio. The homogenate was passed through four layers of gauze, and the filtrate was centrifuged at 15,000× *g* for 20 min. The resulting supernatant was used as an enzyme source.

Catalase activity (CAT, E.C.1.11.1.6) was estimated by the method of Aebi [59]. The reaction mixture contained 50 mM potassium phosphate (pH 7) and 10 mM H_2_O_2_. After the enzyme was added, H_2_O_2_ decomposition was monitored via absorbance at 240 nm (ε = 43.6/(mM cm)).

Ascorbate peroxidase activity (APX, E.C.1.11.1.11) was measured using Nakano and Asada’s method [60]. The reaction mixture contained 50 mM phosphate buffer (pH 7), 1 mM sodium ascorbate, and 25 mM H_2_O_2_. After the addition of the enzymatic extract to the mixture, the reaction was monitored via absorbance at 290 nm (ε = 2.8/(mM cm)).

### 4.6. Statistical Analysis

The results are presented as the means ± SE of three independent experiments, performed sequentially, using duplicate determinations in each experiment. Data were submitted to a two-way ANOVA (inoculation whith *R. irregularis* and CO_2_ level). Pairwise comparisons of means were performed using Turkey’s test, and statistically significant differences were obtained at *p* < 0.05.

## 5. Conclusions

Sunflower plants (*H. annuus L.)* in a CO_2_-enriched atmosphere (eCO_2_) were used to examine the effects of biofertilization with *R. irregularis* (M) on a physiological and metabolic level. This colonization was found to promote plant growth due to an increased nutrient content (P, K, Mg, and B), which favors a greater synthesis of photosynthetic pigments. Biofertilized plants (M) under eCO_2_ conditions had a higher concentration of carbon compounds in their leaves, as compared to non-biofertilized eCO_2_ plants (NM). The biofertilization (M) of sunflowers with *R. irregularis* decreased the C/N ratio as compared to the NM plants, as well as decreasing the hydrogen peroxide content and increasing the antioxidant enzyme activity (catalase and APX). These results suggest that sunflower symbiosis with *R. irregularis* improves the absorption of N, while also decreasing the plant’s oxidative stress. This is relevant for future scenarios in which atmospheric CO_2_ concentrations are expected to increase. Furthermore, it will allow us to verify whether or not biofertilization with *R. irregularis* may potentially replace chemical fertilization in sunflowers, promoting environmentally friendly agricultural practices. This is a further step towards the goal of understanding the mechanisms influencing the C and N dynamic in future scenarios of climate change, where high CO_2_ levels are expected.

## Figures and Tables

**Table 1:** Percentage root colonization and growth parameters of sunflower plants inoculated (M) or not inoculated (NM) with *R. irregularis* (AM) and ambient (a CO_2_) and elevated CO_2_ treatments (eCO_2_). Data are means ±SE. Different letters show significant difference among the treatments according to Tukey’s test (*p* 0.05). ** *p* < 0.01, * *p* < 0.05, *NS* = not significant.

		Root Colonization	Leaf Dry Weight	Stem Dry Weight	Leaf Area
		(%)	(mg plant^−1^)	(mg plant^−1^)	(cm^2^)
aCO_2_					
	NM	ND	940 ± 31.5 ^a^	913 ± 2.6 ^a^	15.9 ± 0.36 ^a^
	M	40.5 ± 0.48 ^b^	1213 ± 36.1 ^b^	1190 ± 23.5 ^b^	17.9 ± 0.44 ^b^
eCO_2_					
	NM	ND	1438 ± 31.7 ^c^	1398 ± 22.6 ^c^	21.85 ± 0.32 ^c^
	M	47.8 ± 0.83 ^d^	1634 ± 40.2 ^d^	1564 ± 24.7 ^d^	25.56 ± 0.28 ^d^
Source of variation					
AM		**	**	**	**
CO_2_		**	*	**	**
AM × CO_2_		**	*NS*	*NS*	*NS*

**Table 2:** Concentration of starch, total soluble sugar (TSS) and proteins (TSP); percentages of carbon (C) and nitrogen (N), C/N ratio in leaves of sunflower inoculated (M) or not inoculated (NM) with *R. irregularis* (AM) and ambient (aCO_2_) and elevated CO_2_ treatments (eCO_2_). Data are means ±SE. Different letters show significant difference among the treatments according to Tukey’s test (*p* 0.05). ** *p* < 0.01, * *p* < 0.05, *NS* = not significant.

		Starch	TSS	TSP	Leaf C	Leaf N	C/N
		(mg g^−1^ DW)	(mg g^−1^ DW)	(mg g^−1^ DW)	(%)	(%)	
aCO_2_							
	NM	30.5 ± 0.53 ^a^	258.2 ± 0.61 ^a^	30.7 ± 0.44 ^a^	39.6 ± 0.61 ^a^	1.42 ± 0.04 ^a^	27.9 ^a^
	M	126.6 ± 1.38 ^b^	261.5 ± 0.41 ^b^	35.6 ± 0.36 ^b^	40.5 ± 0.20 ^a^	1.80 ± 0.44 ^b^	22.5 ^b^
eCO_2_							
	NM	108.7 ± 1.92 ^c^	270.6 ± 0.45 ^c^	31.8 ± 0.49 ^a^	41.9 ± 0.37 ^a^	1.21 ± 0.32 ^c^	34.9 ^c^
	M	308.7 ± 2.24 ^d^	285.7 ± 0.16 ^d^	38.7 ± 0.47 ^d^	40.1 ± 0.45 ^a^	1.45 ± 0.28 ^ad^	27.6 ^ad^
Source of variation							
AM		**	**	**	*NS*	*	**
CO_2_		**	**	**	*NS*	*	**
AM × CO_2_		**	**	*NS*	*NS*	*NS*	*NS*

**Table 3:** P, K (%) B and Fe, B (mg kg^−1^) concentrations in leaves of sunflower inoculated (M) or not inoculated (NM) with *R. irregularis* (AM) and ambient (aCO_2_) and elevated CO_2_ treatments (eCO_2_). Data are means ±SE. Different letters show significant difference among the treatments according to Tukey’s test (*p* 0.05). ** *p* < 0.01, * *p* < 0.05, *NS* = not significant.

		P	K	Mg	Fe	B
		(%)	(%)	(%)	(mg kg^−1^)	(mg kg^−1^)
aCO_2_						
	NM	0.12 ± 0.004 ^a^	0.463 ± 0.008 ^a^	0.18 ± 0.004 ^a^	39.6 ± 0.61 ^a^	12.3 ± 0.20 ^a^
	M	0.20 ± 0.012 ^b^	0.511 ± 0.004 ^b^	0.26 ± 0.008 ^b^	40.5 ± 0.20 ^a^	14.2 ± 0.24 ^b^
eCO_2_						
	NM	0.17 ± 0.008 ^c^	0.494 ± 0.004 ^c^	0.23 ± 0.016 ^c^	41.9 ± 0.36 ^a^	12.9 ± 0.36 ^c^
	M	0.25 ± 0.004 ^d^	0.540 ± 0.024 ^d^	0.31 ± 0.012 ^d^	40.1 ± 0.45 ^a^	17.1 ± 0.20 ^d^
Source of variation						
AM		**	*	*	*NS*	**
CO_2_		**	*	**	*NS*	**
AM × CO_2_		*NS*	*NS*	*NS*	*NS*	*

**Table 4:** Chlorphyll a, chlorophyll b, total Chl content, Chl *a/b* and carotenoids in leaves of sunflower inoculated (M) or not inoculated (NM) with *R. irregularis* (AM) and ambient (aCO_2_) and elevated CO_2_ treatments (eCO_2_. Data are means ± SE. Different letters show significant difference among the treatments according to Tukey’s test (*p* 0.05). ** *p* < 0.01, * *p* < 0.05, *NS* = not significant.

		Chlorophyll *a*	Chorophyll *b*	Total Chl Content	Chl *a/b*	Carotenoids
		(mg g^−1^ DW)	(mg g^−1^ DW)	(mg g^−1^ DW)	Ratio	(mg g^−1^ DW)
aCO_2_						
	NM	5.45 ± 0.03 ^a^	1.50± 0.01 ^a^	6.95 ± 0.04 ^a^	3.63± 0.004 ^a^	1.72± 0.012 ^a^
	M	6.40± 0.04 ^b^	1.89± 0.02 ^b^	8.29 ± 0.060 ^b^	3.38± 0.005 ^b^	2.01± 0.016 ^b^
eCO_2_						
	NM	4.13± 0.03 ^c^	1.08 ± 0.02 ^c^	5.21 ± 0.050 ^c^	3.82± 0.005 ^c^	1.42± 0.008 ^c^
	M	6.65± 0.085 ^d^	1.77 ± 0.01 ^d^	8.42 ± 0.098 ^d^	3.75± 0.013 ^d^	2.44± 0.012 ^d^
Source of variation						
AM		**	**	**	**	**
CO_2_		**	**	**	*NS*	**
AM × CO_2_		*	**	**	*NS*	**
